# Assessing the potential of a *Trichoderma-*based compost activator to hasten the decomposition of incorporated rice straw

**DOI:** 10.1038/s41598-021-03828-1

**Published:** 2022-01-10

**Authors:** Nolissa D. Organo, Shaira Mhel Joy M. Granada, Honey Grace S. Pineda, Joseph M. Sandro, Van Hung Nguyen, Martin Gummert

**Affiliations:** 1 Division of Soil Science, Agricultural Systems Institute, College of Agriculture and Food Science, University of the Philippines Los Baños, Laguna, Philippines; 2 Mechanization and Postharvest Cluster, Sustainable Impact Platform, International Rice Research Institute, 4030 Los Baños, Laguna Philippines

**Keywords:** Environmental sciences, Environmental impact, Environmental microbiology, Fungi

## Abstract

The potential for a *Trichoderma*-based compost activator was tested for *in-situ* rice straw decomposition, under both laboratory and field conditions. Inoculation of *Trichoderma* caused a 50% reduction in the indigenous fungal population after 2 weeks of incubation for both laboratory and field experiments. However, the *Trichoderma* population declined during the latter part of the incubation. Despite the significant reduction in fungal population during the first 2 weeks of incubation, inoculated samples were found to have higher indigenous and total fungal population at the end of the experiments with as much as a 300% increase in the laboratory experiment and 50% during day-21 and day-28 samplings in the field experiment. The laboratory incubation experiment revealed that inoculated samples released an average of 16% higher amounts of CO_2_ compared to uninoculated straw in sterile soil samples. Unsterile soil inoculated with *Trichoderma* released the highest amount of CO_2_ in the laboratory experiment. In the field experiment, improved decomposition was observed in samples inoculated with *Trichoderma* and placed below ground (WTBG). From the initial value of around 35%, the C content in WTBG was down to 28.63% after 42 days of incubation and was the lowest among treatments. This is significantly lower compared with NTBG (No *Trichoderma* placed below ground, 31.1% C), WTSS (With *Trichoderma* placed on soil surface, 33.83% C), and NTSS (No *Trichoderma* placed on soil surface, 34.30% carbon). The WTBG treatment also had the highest N content of 1.1%. The C:N ratio of WTBG was only 26.27, 39.51% lower than the C:N ratio of NTBG, which is 43.43. These results prove that the *Trichoderma*-based inoculant has the potential to hasten the decomposition of incorporated rice straw.

## Introduction

Rice is one of the most important agricultural commodities in the Philippines and other parts of the world, particularly Asia^1^. However, the production of this staple grain to feed the growing population also produces many tons of waste. In 2009, it was reported that 242, 97, 22, and 11 MT of rice straw residue were produced annually in China, India, Thailand, and the Philippines, respectively^2^. To eliminate this residue, one cost-effective method that is widely practiced is burning rice straw in the field^2^. In the Philippines alone, 95% of the residue undergoes open-field burning^2^. Rice straw burning is widely employed because it is less laborious compared to other practices such as straw incorporation into the soil^3^. Burning can also eliminate many pathogens^4^, and, most importantly, it results in rapid and complete residue removal^5^. While burning seems to be the most practical way of removing the straw, this practice has several undesirable effects including loss of major nutrients^6^. The nutrient loss caused by straw burning can reach 80% for nitrogen (N)^7^, 25% for phosphorus (P)^8^, 21% for 33 potassium (K)^9^, and 4–60% for sulfur (S)^10^. In addition, biomass burning is the second-largest source of trace gases and the largest source of primary fine carbonaceous particles in the global troposphere^11–14^. A recent Life Cycle Assessment (LCA) of existing in-field and off-field rice straw management practices reported that rice cultivation with in-field burning is the worst option with the lowest energy efficiency and highest air pollution emission^15^.

The use of modern farm equipment enables farmers to easily manage farm operations. For instance, the combine harvester combines harvesting operations—reaping, threshing, and winnowing into a single process. The use of combine harvesters has therefore been used for rice and has expanded rapidly worldwide and major producers, such as Vietnam and Cambodia, almost exclusively rely on them^16^. Because of this technology, harvesting can be performed even with minimal manpower and the chopped rice straw can be incorporated into the soil for decomposition.

Rice straw incorporation can solve environmental pollution caused by straw burning. In a meta-analysis in China which compared the effects of straw burning and incorporation on the Net Global Warming Potential (NGWP) to include C sequestration, it was found that switching from burning to straw incorporation could mitigate 34.18 Mt. CO_2_ eq year^−1^ or 31% of total emissions in the country^17–19^. Studies show that early incorporation is one the most cost-effective, climate-smart rice straw management options^19, 20^. High rates of straw incorporation under aerobic conditions can sequester soil organic carbon (SOC) with a minimal increase in emissions compared to incorporation under flooded conditions. Practices that optimize SOC sequestration while minimizing emissions, such as early straw incorporation with alternate wetting and drying water management, could be an important step towards carbon–neutral rice systems^19^. Straw incorporation has been shown to enhance nutrient recycling and provide soil fertility benefits by increasing SOC and yields of subsequent crops^8, 21–24^. The incorporated straw can also provide substrates to promote biodiversity through flourishing of invertebrates that decompose the straw, which in turn enhances nutrient cycling in paddy soils^24, 25^. A recent study in Vietnam showed that the addition of rice straw improved soil pH, soil organic C, and nutrient content compared to addition of ash from burned straw^26^. Due to the low N content in straw, however, large quantities would be needed to supply adequate amounts of N. In addition, the straw has to decompose before the nutrients can become available for uptake and the rate of decomposition and supply of nutrients depend on soil type and season. A sufficient level of decomposition is also needed for mechanized seedbed preparation to prevent clogging of seeders.

Unfortunately, rice straw decomposition takes time. Rice straw is composed mainly of cellulose, hemicellulose, and lignin^27, 28^. Silica, polysaccharides, and lignin were reported to form complex rigid structures that inhibit the decomposition of straw lignin^28–30^. In a recently reported 5-year experiment, the release of C, N, cellulose, and hemicellulose was found to occur mainly during the first and second years after straw incorporation. Moreover, the release of P and K occurred mainly during the first month, while lignin was released at various rates throughout the entire study period^31^. Long-term incorporation of crop residues in flooded rice soil can increase soil organic matter, total N, and soil biological activity^22^. Continuous incorporation of crop residues after each crop can eventually increase the N-supplying capacity of rice soils^32^. In a study in Vietnam, soil N increased from 0.65 to 0.85% following 9 years of cropping with incorporation of rice straw while straw removal caused a decline in soil N^33^. However, the benefits of incorporated residues on soil organic matter and soil N supply seldom translate into increased yield or profit for flooded rice^5^. The production of two or three rice crops annually also results in the production of large quantities of straw, with little turnaround time between crops. This results in limited decomposition of the straw when incorporated, with potential negative effects on nutrient availability and use efficiency of applied fertilizers for the subsequent crop^21, 22, 24^.

Because of the environmental impacts of straw burning and the importance of quick turnaround times between crops, there is a need to hasten the decomposition of rice straw that is incorporated into the field. Accelerating the decomposition process can promote rice straw incorporation as a preferred practice compared to burning and the application of microbial inoculants could possibly help in doing so.

Microorganisms play a role in the decomposition process as they are the ones responsible for the conversion of crop residues to soil organic matter. Rice straw decomposition is a complex process made up of several subprocesses and involves a diversity of organisms. One of the most common rice straw-degrading microorganisms are the fungi *Trichoderma*^34^. *Trichoderma* spp. play an important role in biological decomposition and are also known to produce cellulolytic and hemicellulolytic enzymes. *Trichoderma* spp. are filamentous soil fungi that secrete a well-balanced cellulolytic complex, which efficiently hydrolyzes cellulosic substrates into monomeric glucose chain. The potential of *Trichoderma* spp. in hastening the decomposition of straw has been previously reported^35^. In the Philippines, the inoculation of a straw-based compost pile with *Trichoderma harzianum* was reported to have a composting time of less than half of the conventional methods of composting^36, 37^. *Trichoderma* spp. are also reported to be able to degrade agricultural and domestic waste relatively quickly without emitting bad odors^38^. Among the many species of *Trichoderma, T. harzianum* was reported to produce the highest amounts of cellulolytic enzymes that are useful in degrading straw^39^. *T. viride* was also reported to produce ligninolytic enzymes that were able to reduce the lignin content of rice straw^40^. The use of *Trichoderma-*based composting technologies has been widely accepted and may have a potential to enhance in situ rice straw decomposition. However, there is limited knowledge on the potential application of *Trichoderma* spp. to enhance the decomposition of rice straw that is incorporated into the field.

This study aimed to determine if a commercially available *Trichoderma*-based compost activator, which is usually applied in composing piles, can be used to hasten the decomposition of rice straw that is incorporated into the field.

## Materials and methods

Two sets of experiments were performed to determine the potential of a *Trichoderma* inoculant for in situ rice straw decomposition. The first one is a laboratory incubation experiment that determines the effect of *Trichoderma* on the decomposition rate of rice straw in sterile and unsterile soils. The second is a field experiment that analyzed the effect of *Trichoderma* application and straw placement on in situ rice straw decomposition using the litterbag technique. The study complies with local and national guidelines.

### Laboratory incubation experiment

The incubation experiment was conducted at the Soil Microbiology Laboratory, Division of Soil Science, Agricultural Systems Institute, College of Agriculture and Food Science of the University of the Philippines Los Baños (UPLB) to determine the effect of *Trichoderma* on the decomposition of rice straw mixed with sterile and unsterile soil. The experiment was laid in a completely randomized design with five treatments, three replicates per treatment, and nine sampling periods.

Freshly harvested rice straw was collected from the Zeigler Experimental Station (ZES), IRRI, Los Baños, Laguna. It was air-dried and chopped prior to the set-up of the experiment. The treatments included: unsterile soil only (USO), unsterile soil plus rice straw (USRS), unsterile soil plus rice straw plus *Trichoderma* (USRST), sterile soil plus rice straw (SSRS), and sterile soil plus rice straw plus *Trichoderma* (SSRST). Fifty grams of soil, 5 g of rice straw, and 500 mg of *Trichoderma* were used in the designated treatments. The treatments were placed in a wide-mouth incubation jar and mixed thoroughly. Water was added to keep the rice straw moist, but not saturated. A CO_2_ trap consisting of 30 mL of 0.3 N NaOH was placed and the jar was screwed tightly and incubated at room temperature. Three jars were taken from each treatment at 1, 3, 7, 14, 21, 28, and 42 days after the incubation for the determination of CO_2_ evolved. The *Trichoderma* and fungal population were also determined for samples taken at 7, 14, 28, and 42 days after incubation.

### Field incubation experiment

The field incubation experiment tested the effect of *Trichoderma* application and straw placement on the decomposition of rice straw under field conditions. The experiment was laid out in a completely randomized design with two factors, four treatments with three replicates per treatment, for five sampling periods. The two factors considered are presence or absence of the *Trichoderma* activator and placement of the litterbag (soil surface or below ground). Hence, the treatments are: no *Trichoderma* on soil surface (NTSS), no *Trichoderma* below ground (NTBG), with *Trichoderma* on soil surface (WTSS), and with *Trichoderma* below ground (WTBG).

The field experiment used the litter bag technique in analyzing the effect of treatments on rice straw decomposition. To perform this, freshly harvested rice straw was collected from the ZES. A total of 100 g of air-dried and chopped rice straw were placed in nylon bags with a mesh size of 20 × 20 µm each. The mesh size selected for this experiment was very small to ensure that soil animals did not enter the litterbag and affect the decomposition as previously reported^25^. One gram of *Trichoderma* activator was added to the litter bags with the *Trichoderma* treatment. A total of 96 litterbags were placed in the field, half of which were inoculated with *Trichoderma*. One set of the treatments was placed at the soil surface secured with steel wire while the other set was buried 10 cm below ground. Six litterbags per treatment were sampled at 7, 14, 21, 28 and 42 days after the field set-up. One set of three replicates was subjected to weight loss determination, while the other three replicates were used for the determination of fungal populations (*Trichoderma*, other fungi, and total fungal populations) and analysis for total carbon and total nitrogen.

### Data collection

The analyses performed on the rice straw and soil samples are discussed in detail below.

#### Determination of CO_2_ evolved using the titration method

The contents of the CO_2_ trap were transferred to a 125-mL Erlenmeyer flask. Two to three drops of phenolphthalein indicator and 1.0 mL of 50% BaCl_2_ were added and titration was performed using 0.2 N HCl until the pink color just disappeared. The amount of acid used to neutralize the base was recorded. Likewise, 30 mL of a fresh sample of 0.3 N NaOH were titrated. The amount of CO_2_ evolved per 100 g rice straw was calculated using the following formula:

$${\text{mg of}}\, {\text{CO}}_{2} = \left( {B - V } \right) \times 22 \times N \times 2$$

where:

*V* = Volume (mL) of acid used to titrate the alkali in the CO_2_ trap to the end point.

*B* = Volume (mL) of acid used to titrate the fresh sample of alkali to the end point.

*N* = Normality of the acid.

22 = Weight of 1 meq CO_2_ in mg.

#### Determination of fungal populations

The fungal populations (*Trichoderma,* other fungi, and total fungal population) were determined using the spread-plate method. Ten grams of sample from the incubation experiment were placed in 95 mL of sterile distilled water and were serially diluted. One hundred microliters of liquid from dilutions 10^–3^ to 10^–5^ were spread-plated on Potato Dextrose Agar (PDA) with Rose Bengal supplemented with 50 mg L^−1^ streptomycin. Potato dextrose agar (PDA) is an enrichment medium that enables the growth of all fungal species present from the sample. *Trichoderma* colonies can be differentiated from other fungi because they have irregular shape, with cottony and yellowish green conidia with white mycelia towards the margin^41^. They also have a yellow irregular zone surrounding the colony at the bottom side of the glass plates. Similarly, 10 g of sample were oven-dried at 105 °C for 72 h. The colonies that formed were counted and the populations were calculated using the formula:

$${\text{Log}}\frac{CFU}{g} =\frac{{Ave.\,\#\,{\text{of colonies on best dilution }} \times {\text{ dilution factor}}}}{{{\text{Oven}} - {\text{dry weight of soil sample}}}}$$

#### Determination of total C and total N (combustion method)

Rice straw samples were ground twice using Wiley and Ball Mill grinders until the straw had the desired talc-like consistency. A small amount (1.5 to 2.0 g) of the powdered samples was submitted to the IRRI Analytical Service Laboratory for total C analysis using a dynamic combustion system coupled with a gas chromatograph equipped with a thermal conductivity detector (GC-TCD).

#### Determination of weight loss and decomposition rate

The retrieved litterbags were separated from the adhering soil particles by washing them carefully with tap water and then pat dried using a clean cloth. The straw was removed from the litterbags and weighed to determine its fresh weight. Next, the straw was oven-dried at 70 °C for 72 h and then weighed again for its oven-dried weight. The decomposition rate of the straw was determined based on mass loss using the exponential decay model^42^^:^

$$M_{t} = M_{o} e^{ - kt}$$

where:

*k* = rate of decomposition.

*M*_*o*_ = initial dry mass of rice straw.

*M*_*t*_ = dry mass of rice straw after t time.

*t* = time (days) the straw is in the soil.

### Statistical analysis

The analysis of variance (ANOVA) for completely randomized design (CRD) was performed on the collected data per sampling period. Least Significant Difference (LSD) was used in comparing the means of all parameters measured using the Statistical Tool for Agricultural Research (STAR) software.

## Results

### Characterization of the soil and environment

**Figure 1:**
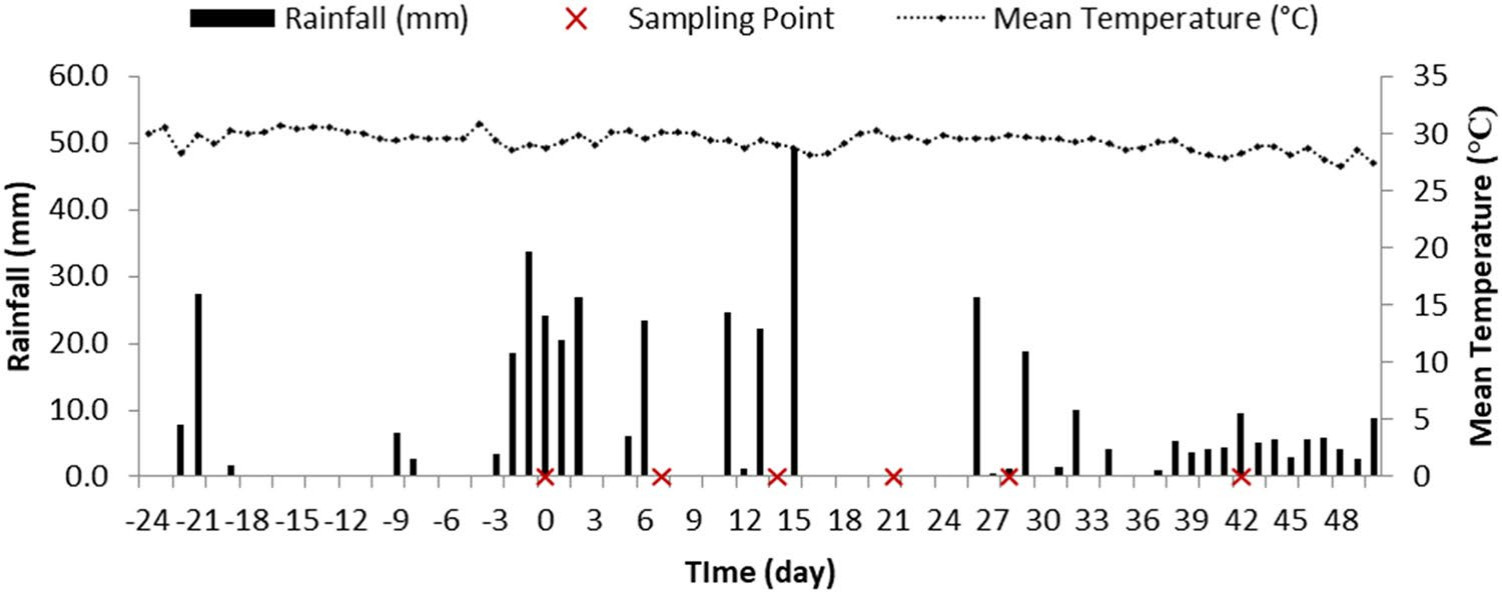
Average daily rainfall and temperature in the field during the experiment period.

ZES at IRRI has 200 ha of research fields dedicated to different rice research activities. One of the fields (plot UJ) was selected as the study site due to its availability during the time of the study. The site was dried during its fallow period and was previously planted with lowland rice. Plot UJ is situated at 14° 08′ 40.48″ N and 121° 15′ 53.21. Soil samples collected from plot UJ were found to have a loam texture, with 23.33% clay, 43.33% silt, and 33.33% sand. The soil also has a neutral pH of 7.04. The laboratory incubation experiment was exposed at an ambient temperature of around 27 °C. The average daily temperature during the field incubation was a bit higher and ranged from 29.1 to 29.6 °C (Fig. 1). Higher precipitation was observed during the start of the field incubation experiment, with daily rainfall of around 20 to 30 mm. Lesser amounts of rainfall were observed at the end of the first week, while the second week of incubation was characterized by higher precipitation at the later part, around 11 to 14 days. The highest precipitation of almost 50 mm was observed on the first day of the third week, but no precipitation was observed afterwards until the last few days of the fourth week. This was followed by steady precipitation until the last sampling (Fig. 1).

### Fungal population dynamics in decomposing straw under laboratory and field conditions

**Figure 2:**
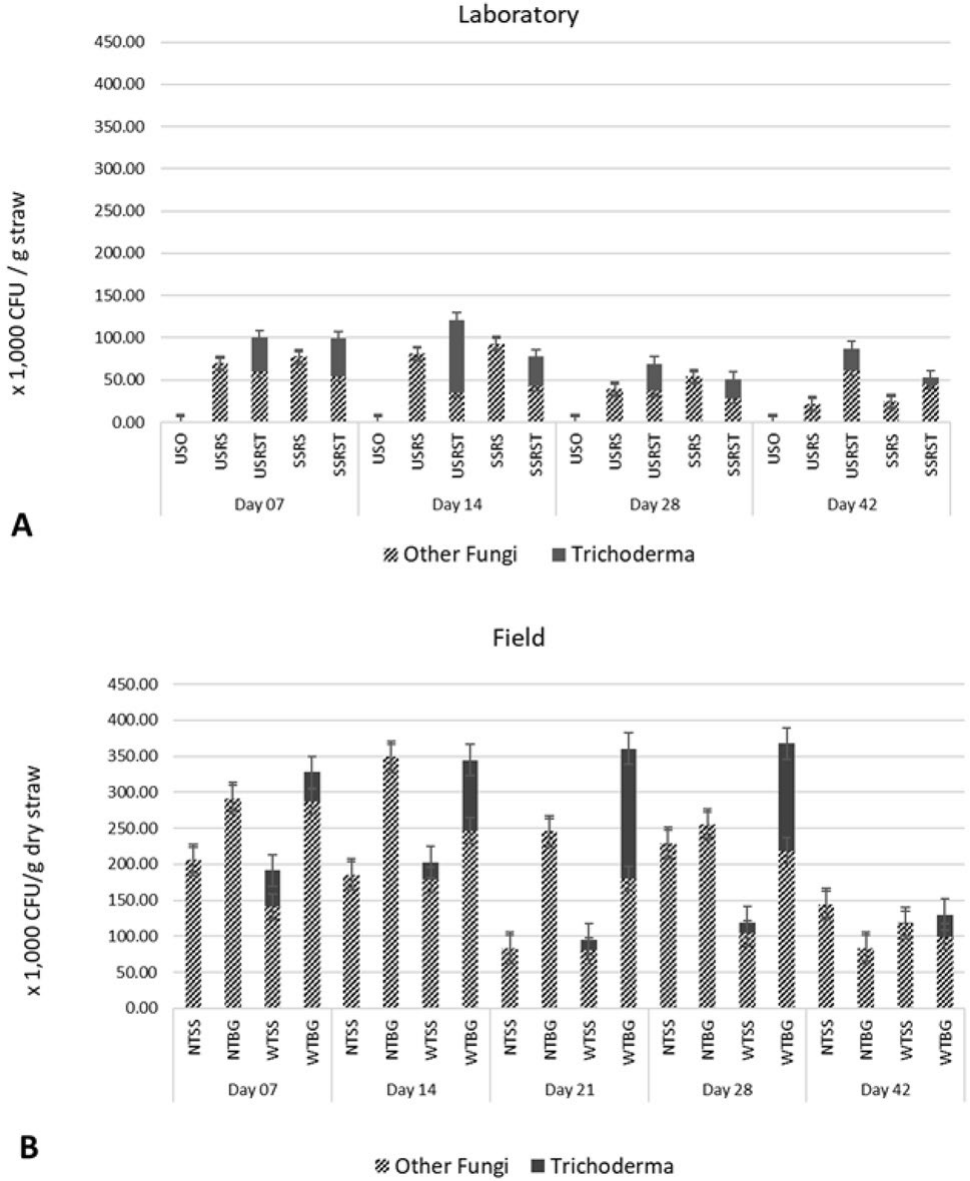
Fungal population dynamics in the (**A**) laboratory and (**B**) field incubation experiments. USO = unsterile soil only; USRS = unsterile soil plus rice straw; USRST = unsterile soil plus rice straw plus *Trichoderma*; SSRS = sterile soil plus rice straw; SSRST = sterile soil plus rice straw plus *Trichoderma*; NTSS = no *Trichoderma* on soil surface; NTBG = no *Trichoderma* below ground; WTSS = with *Trichoderma* on soil surface; WTBG= with *Trichoderma* below ground.

Under both laboratory and field conditions, *Trichoderma* was only observed in inoculated rice straw (Fig. 2A and B). This indicated that the inoculation of *Trichoderma* was successful and that the *Trichoderma* did not contaminate the uninoculated samples. Under laboratory conditions, no fungal population was detected in the unsterile soil sample (USO treatment). This indicates that the fungal population observed mainly comes from the rice straw and *Trichoderma* that was added to the rice straw-soil mixture. From a population of a 4 × 10^4^ CFU/g sample after the first week of incubation, the *Trichoderma* population in the USRST treatment was doubled to 8 × 10^4^ after 14 days. On the other hand, the *Trichoderma* population in SSRST treatment was reduced to 3.4 × 10^4^. The *Trichoderma* population declined at days 28 and 42, with the USRST consistently having the highest *Trichoderma* population. Populations of other fungi were lower in the *Trichoderma*-treated samples than the uninoculated counterparts during the first 4 weeks (7, 14, and 28 days) of incubation. A significant effect of the inoculation was found at day 14, in which the other fungal populations in the USRS were 8.12 × 10^4^ while USRST had 3.52 × 10^4^. The obtained values indicated a 56.7% reduction of fungal populations brought about by *Trichoderma* inoculation. The same was observed in sterile samples in which SSRS had 9.3 × 10^4^ while USRST had a population of 4.32 × 10^4^ (53.55%). Further reduction in population was observed at day 28, but the reduction of the populations of other fungi was reduced to 6.75 and 46.98% in USRST and SSRST, respectively. *Trichoderma* was found to dominate the inoculated samples during the first 4 weeks of incubation. The fungal populations of both the USRST and SSRST treatments had around 40% *Trichoderma* after 1 week of incubation. At day 14, a spike in the *Trichoderma* population was observed in USRST, wherein 70% of the total fungal population consisted of *Trichoderma*. SSRST, on the other hand, maintained the 40% *Trichoderma* population until day 28. At day 28, the *Trichoderma* population in USRST was reduced to around 40% of the total fungal population. The *Trichoderma* population in both treatments was further reduced at day 42. The reduction in the *Trichoderma* population was simultaneous with the increase in the population of other types of fungi. Although inoculated treatments had lower populations of other fungi at the initial stages of the experiment, *Trichoderma*-treated samples had a higher total and other fungal populations compared to the uninoculated counterpart at 42 days. Populations of other fungi in USRST at day 42 were 6.13 × 10^4^, 185% higher than USRS, which had other fungal populations of only 2.15 × 10^4^.

The increase in total fungal population reached up to 300%, with USRS having 2.15 × 10^4^ and USRST having 8.75 × 10^4^. Similar results were obtained in sterile soil samples, with 87 and 115% increases in other fungi and total fungal populations, respectively.

The trends observed in the laboratory experiment were also observed in the field. The *Trichoderma* population increased at days 14, 21, and 28, and eventually declined at day 42. A significant effect of placement was observed wherein buried litterbags had significantly higher *Trichoderma* and total fungal population compared to the litterbags placed on the surface (Table 1; Fig. 2B). The *Trichoderma* population in WTBG increased from 4.03 × 10^4^ at day 7 to 9.78 × 10^4^ at day 14 (a 142% increase) and further increased to 1.80 × 10^5^ at day 21, which was the highest observed throughout the incubation period. The *Trichoderma* population slightly declined on day 28, with a population of 1.5 × 10^5^ and further after 42 days, with a population of only 3.03 × 10^4^. The same trend was observed with the indigenous fungal populations, in which inoculated samples had lower indigenous fungal populations compared with the uninoculated counterpart. The effect, however, was not as drastic as the effect observed in the laboratory. A reduction of 29.30% (3.49 × 10^5^ in NTBG, 2.46 × 10^5^ in WTBG) and 26.69% (2.45 × 10^5^ in NTBG, 1.89 × 10^5^ in WTBG) was observed at days 14 and 21, respectively. The effect of placement on the populations of other fungi was also significant at day 21, with the litterbags placed below ground having higher other fungal populations (Table 1). At day 42, the other fungal populations in WTBG (9.98 × 10^4^) were slightly higher than that of NTBG (8.35 × 10^4^) but was not significantly different. The total fungal population (*Trichoderma* + other fungi) was significantly affected by the placement method at days 14, 21, and 28 (Table 1). Higher total fungal population was consistently observed in the WTBG treatments where the litterbags were inoculated with *Trichoderma* and placed belowground (Fig. 2B).

**Table 1:** Effect of placement on the fungal populations in the litterbags for each sampling period.

*Fungal Population* (×1000 CFU/g straw)*	Treatment	Day 7	Day 14	Day 21	Day 28	Day 42
*Trichoderma*	Below Ground	40.40^a^	97.83^a^	180.00^a^	149.17^a^	30.30^a^
	Soil Surface	50.40^a^	24.40^b^	15.32^b^	15.55^b^	0.25^a^
Other Fungi	Below Ground	289.42^a^	297.82^a^	212.83^a^	236.83^a^	91.62^a^
	Soil Surface	173.42^a^	182.13^a^	81.92^b^	166.50^a^	131.07^a^
Total Fungi	Below Ground	309.33a	346.67^a^	302.83^a^	311.50^a^	106.70^a^
	Soil Surface	198.67a	194.50^b^	89.42^b^	174.23^b^	131.07^a^

*Means with the same letter are not significantly different (*p* < 0.05).

### Observation of decomposition through CO_2_ evolved from the decomposing rice straw in the laboratory experiment

Because the liberation of CO_2_ occurs during decomposition, CO_2_ production is a widely accepted quantitative expression of the decay process^43^. In the laboratory experiment, it was observed that treatments inoculated with *Trichoderma,* USRST and SSRST, consistently released higher amounts of CO_2_ compared to their uninoculated counterparts, USRS and SSRS, respectively (Table 2). The highest amount of CO_2_ evolved was observed in the SSRST treatment with sterile soil and *Trichoderma* inoculant at days 1, 3, and 7. The SSRST treatment consistently had significantly higher amounts of CO_2_ evolved compared with SSRS during the first 4 weeks of incubation (*p* < 0.05). At day 1, SSRST released 201.23 mg CO_2_. This is 35% compared to SSRS, which released only 149 mg CO_2_. In the succeeding sampling periods, SSRST consistently released higher amounts of CO_2_, around 16% higher than SSRS on the average. The effect of *Trichoderma* inoculation was more pronounced in sterile soil samples than unsterile soil samples for the first 4 weeks of incubation. A significant effect on soil condition and inoculation was observed at day 21, in which USRST (unsterile soil + rice straw + *Trichoderma*) released the highest amount of CO_2_. At day 42, simultaneous to the decline in fungal population, the effect of inoculation on CO_2_ production was no longer significant. Having the highest total fungal population at the end of the 42-day incubation, the USRST treatment (unsterile soil + rice straw + *Trichoderma* inoculant) also had the highest amount of CO_2_ evolved at 2943.16 mg CO_2_, followed by USRS (unsterile soil without *Trichoderma*) with a value of 2763.64 mg CO_2_ and SSRST (sterile soil + rice straw + *Trichoderma* inoculant), which released a total of 2,405.04 mg CO_2_.

**Table 2:** Effect of Soil Sterility and *Trichoderma* Inoculation on the Cumulative CO_2_ evolved (mg CO_2_) from the decomposing rice straw for each sampling period.

Treatment	Day 01	Day 03	Day 07	Day 14	Day 21	Day 28	Day 42
USO	29.63^d^	96.51^d^	91.23^c^	44.88^c^	− 84.48^d^	− 209.44^c^	− 148.72^c^
USRS	177.17^b^	497.49^b^	758.27^a^	891.00^a^	1037.96^b^	1156.76^a^	1551.44^a^
USRST	186.56^ab^	503.36^ab^	793.17^a^	945.56^a^	1123.32^a^	1274.68^a^	1593.24^a^
SSRS	149.01^c^	443.52^c^	688.45^b^	803.15^b^	889.39^c^	1016.99^b^	1315.31^b^
SSRST	201.23^a^	515.68^a^	802.27^a^	938.96^a^	1032.24^b^	1165.12^a^	1514.48^ab^

*Means with the same letter are not significantly different (*p* < 0.05).

USO = unsterile soil only; USRS = unsterile soil plus rice straw; USRST = unsterile soil plus rice straw plus *Trichoderma*; SSRS = sterile soil plus rice straw; SSRST = sterile soil plus rice straw plus *Trichoderma.*

### Field experiment

#### Effect of *Trichoderma* inoculation and placement method on the percent weight loss and decomposition rate (k) of rice straw

While *Trichoderma* inoculation resulted in higher decomposition rates in the laboratory experiment, the field experiment seems to show otherwise. Lower percent mass loss and decomposition rates were observed in treatments inoculated with *Trichoderma* (WTSS and WTBG) compared to those without *Trichoderma* (NTSS and NTBG). At day 21, when the highest *Trichoderma* population was observed in the WTBG treatment, the mass loss in NTSS and NTBG was significantly higher at 32.20 and 37.67%, respectively, while inoculated treatments WTSS and WTBG only resulted in 20.06 and 28.29% mass losses, respectively. The highest percent mass loss value of 38.99% was observed in the NTBG treatment at day 28, while inoculated samples WTSS and WTBG were significantly lower at only 23.69 and 24.01%, respectively. At day 42, a decrease in mass loss was observed in NTBG (37.83%) while the mass loss in WTBG increased to 31.23%. At the end of the 6-week incubation, the percent mass loss in WTBG was comparable with NTSS and NTBG.

#### Effect of *Trichoderma* inoculation and straw placement on C and N contents and C:N ratio

No significant difference was observed in the C content of litterbags during the first 3 weeks of incubation. Significant effects of placement and *Trichoderma* application were observed at days 28 and 42, in which the WTBG treatment had the lowest C content of 29.77% compared to 34.30% in NTSS, 31.10% in NTBG, and 33.83% in WTSS. The N content was not significantly different among the treatments during the first 4 weeks of incubation. At day 42, WTBG had the highest N content of 1.1%, resulting in the lowest C:N ratio compared to the other treatments. The C:N ratio of WTBG was only 26.27—39.51% lower than the C:N ratio of NTBG, which was 43.43.

## Discussion

### Effect of inoculation on *Trichoderma* and total fungal population in the field and laboratory

For both the laboratory and field experiments, the *Trichoderma* population was significantly higher in inoculated samples (*p* < 0.01), indicating that no contamination occurred during the course of the study. Since the environmental conditions in the laboratory, such as temperature and moisture, were constant, the fungal population dynamics observed is a reflection of the microbial interactions that occurred in the samples. At the start of the laboratory incubation, there was a significant reduction of the indigenous fungal populations in inoculated samples. *Trichoderma* spp. are active ingredients in many commercial bio-fungicides used to control a range of economically important fungal plant pathogens^44^. *Trichoderma* was reported to control soilborne pathogens, which are mostly fungi, such as *Fusarium*, *Rhizoctonia*, and *Pythium*^45^. The antagonistic activity of the *Trichoderma* strains is attributable to complex mechanisms, including the activity of cell wall-degrading enzymes, direct competition for nutrients, antibiosis, and the induction of plant defense responses at both the local and systemic levels^46^. *Trichoderma* attaches to the host fungi and then coils its hyphae around the host, forms appressoria on the host surface, penetrates the host cell, and collapses the host hyphae^47^. The inoculant applied may have used the indigenous fungal populations as hosts and this resulted in the increase in the *Trichoderma* population and simultaneous reduction in the populations of other fungi.

Fungal succession was reported to be related to substrate quality This has been well documented in studies on decomposition^48–52^. In the laboratory incubation experiment performed in this study, limited amounts of substrates are available because rice straw is highly cellulolytic and ligninolytic. In cases like this, extracellular enzymes, such as cellulases, play a key role in decomposing complex organic polymers into oligomeric and monomeric substances that are small enough to be transported inside the cell^43, 53^. Because of the known ability of *Trichoderma* to produce high amounts of cellulolytic and hemicellulolytic enzymes, it can be assumed that inoculated samples have higher amounts of cellulases compared to uninoculated ones. This will enhance the hydrolysis of the cellulosic substrates in the rice straw and eventually lead to the production of higher amounts of monomeric glucose units that can be taken up by the indigenous fungal populations. The increased amount and quality of substrates available in inoculated samples can lead to the promotion of microbial growth. This was reflected during the last part of the experiment wherein the total fungal population was significantly higher in inoculated samples (p < 0.05). Similarly, the continuous decline of the fungal populations in uninoculated samples can be attributed to the depletion of the readily available substrates because the microorganisms present are not capable of producing cellulolytic enzymes that can degrade the complex cellulosic substrates.

Independent and combined effects of *Trichoderma* inoculation and the method of straw placement on the fungal populations were observed during the last 2 weeks of field incubation. A higher *Trichoderma* population was observed in inoculated samples that were placed below ground than those placed on the surface (Table 1, *p* < 0.01). The significantly higher *Trichoderma* in the WTBG over the WTSS could be explained by the difference in soil moisture and the degree of contact between rice straw and soil as affected by the method of straw placement. Buried litterbags are expected to have higher moisture since the moisture of surface-applied litterbags may dry up easily during warm days^54^. This is consistent with previous research that reported greater fungal colonization in litterbags with intimate litter-soil contact^55^. A consistent trend was observed between the laboratory and field experiments, in which the *Trichoderma* population starts to decline at the fourth week of incubation. It is interesting to note that, while the indigenous fungal populations initially declined in inoculated samples during the first 2 weeks of incubation, inoculated samples were the ones with a higher total fungal population at the end of the incubation period. The total fungal population in the USRST (8.75 × 10^4^) was 300% higher than the USRS (2.15 × 10^4^), while the total fungal population in the WTBG (1.30 × 10^5^) was 55.66% higher than the NTBG (8.35 × 10^4^).

### Effect of ***Trichoderma*** inoculation on CO_2_ evolution

The overall process of decomposition generally involves a broad spectrum of complementary microbes that act in concert on a substrate^43^. These microorganisms produce different kinds of enzymes that act on the organic compounds present. Soil microbes use residue components as substrates for energy and as carbon sources in the synthesis of new cells. Energy is furnished to the microbial cells through the oxidation of the organic compounds and a major product is CO_2_, which is released back into the atmosphere. Microorganisms are generally short-lived and themselves are decomposed by successive populations that find the dying cells to be more suitable substrates than the initial residues^43^. Given this principle, we can assume that the C present in the rice straw has three faiths: (1) released as CO_2_; (2) converted into microbial biomass, whether bacterial or fungal; or (3) remaining as an undecomposed component of the straw.

In the laboratory experiment, the use of sterile or unsterile soil was intentionally done to consider the influence of the soil’s indigenous microbial populations on rice straw decomposition. The SSRST treatment released the highest amount of CO_2_ during the first week of incubation. The amount released was 35% more compared to its uninoculated counterpart (SSRS) on day 1, suggesting that *Trichoderma* contributed greatly to the decomposition of the substances present in the soil. The high amounts of CO_2_ released during day 1 were an indication that most of the C present was being used by the microorganisms (composed mainly of *Trichoderma*) for respiration to produce energy that will be used for metabolic activities during the period of adjustment to the new environment (the incubation jar). The contribution of *Trichoderma* to CO_2_ evolution lowered to around 16% in the succeeding days, but the difference in CO_2_ evolved between inoculated (SSRST) and uninoculated (SSRS) samples were still significantly different until day 28 (Table 2).

At this point, we can assume that some of the C was now being used as part of the microbial biomass, especially since *Trichoderma* produces hyphae that branch into thick mycelia. Microbial efficiency is the term used for the percentage of C that is being converted into microbial biomass^43^. In unsterile soil samples, the amount of CO_2_ evolved in inoculated samples (USRST) was also higher than the CO_2_ evolved in uninoculated ones during the first 2 weeks of incubation. The difference, however, was not significant and this was possibly caused by the indigenous microorganisms that are present in the unsterile soil. While some groups of microorganisms are metabolizing and releasing CO_2_, another group may be using the C to build microbial biomass. Microbial interactions are also most likely to occur, and this complex process may have influenced the release of CO_2_. A significant effect of inoculation on CO_2_ evolution was observed on day 21. From day 14 onwards, the USRST treatment (unsterile soil + *Trichoderma* inoculant) had the highest amount of CO_2_ evolved among all the samples. This was followed by SSRST (sterile soil with *Trichoderma*) and USRS (unsterile soil without *Trichoderma*) treatments. These observations indicate that the *Trichoderma* inoculant played a big role during the first 3 weeks of incubation.

### Effect of *Trichoderma* inoculation on % mass loss and decomposition rate

*Trichoderma* has been reported to be capable of degrading straw and other organic materials^38, 40, 56–60^. The success in using *Trichoderma* for composting has been well-documented. The inoculation of straw-based compost piles with *Trichoderma harzianum* was reported to have a composting time of less than half of the conventional methods of composting^36, 37^. Rice straw treated with *Trichoderma harzianum* and cow dung slurry was reported to increase the population of beneficial microorganisms and improved crop yield^61^. The decomposition of empty fruit bunches (EFB) and palm oil mill effluent (POME) was also reported to be reduced from 4 to 6 months to 21–45 days^62^. In vitro and in vivo conversion of solid organic waste was also reported possible using *Trichoderma*^59^. Commercially available *Trichoderma*-based inoculants are currently available in the market and most of these products are successfully used in composting.

The results of the laboratory experiment in this research supported the previous claims where inoculated samples release higher amounts of CO_2_. In the field experiment, however, rice straw samples inoculated with *Trichoderma* were the ones with the low percent mass loss and decomposition rate throughout the incubation period. The highest percent mass loss value of 38.99% was observed in NTBG treatment at day 28, while inoculated samples WTSS and WTBG were significantly lower at only 23.69 and 24.01%, respectively. While most research considers mass loss as an indicator of decomposition, this may not be applicable in this study because of the proliferation of fungi in the inoculated litterbags, which resulted in an increase in biomass. Recall that the WTBG treatment had the highest *Trichoderma* and total fungal population with sampling at both days 21 and 28. During these sampling days, the percent mass loss values of *Trichoderma*-treated samples were significantly lower than the uninoculated ones, possibly because of the increased biomass from the high fungal population. At day 42, simultaneous to the decrease in total fungal population in WTBG, the percent mass loss increased to 31.23% and was no longer significantly different with NTBG, which had a mass loss of 37.83%. Since the increase in mass brought about by the growth of *Trichoderma* and other fungi was not accounted for, the measurement of decomposition rate through mass loss and exponential decay model cannot accurately represented the rate of decomposition that occurred. A previous study reported increased mass loss in buried rice straw with the use of *Trichoderma* and frequent watering^58^. However, upon checking, that research only used 5 g of rice straw, which is 20 times smaller than the initial amount of straw used in this experiment. The previous research also mentioned that watering daily resulted in increased mass loss, but further details were provided in the methodology. Fungal biomass varies widely within and across biomes in relation to litter composition, root density, and nutrient availability. Fungi may comprise of up to 20% of the mass of decomposing plant litter and the fungal mycelia grow radially as fractal networks in soil, wood, and litter^63, 64^ Since determining the fungal biomass was not done in this study, we cannot be sure of the actual proportion of biomass from the fungi and from the rice straw.

### Effect of *Trichoderma* inoculation on C and N content and C:N ratio of decomposing rice straw

As mentioned above, C present in the rice straw has three faiths: (1) released as CO_2_; (2) converted into microbial biomass, whether bacteria or fungi; or (3) remaining as an undecomposed component of the straw. Since the CO_2_ is released back into the atmosphere, the C present in the litterbags represented C from both the microbial biomass and undecomposed rice straw component. At the start of the experiment, the C content in all the treatments was 34 or 35%. During this period, the total fungal population was also similar among the treatments. While the mass gain brought about by the increased *Trichoderma* and total fungal population cannot be determined, a comparison of the fungal population and C content of the WTBG and NTBG treatments can help explain the relevance of high fungal population on the mass and C content of the samples. After 3 weeks of incubation, the C content of uninoculated samples, NTBG and NTSS, was lower than the initial value by about 2%, while the C content of WTBG was still close to the initial value (Table 3). During the same period, the WTBG treatment had the highest *Trichoderma* population among the treatments, (*p* < 0.01). Since the other treatments did not have a significantly high *Trichoderma* and total fungal population, we can assume that the C in the NTBG, NTSS, and WTSS was mainly in the form of undecomposed rice straw. On the other hand, we can also assume that a significant amount of C measured in WTBG treatment came from both the undecomposed straw and the fungal biomass present in the litterbags. At day 28, despite having the highest total fungal population, the C content in the WTBG treatment decreased to 29% from an initial value of 34.5%. This was significantly lower compared with NTBG (31.1% C), WTSS (33.83% C), and NTSS (34.30% C) (*p* < 0.05). Since the total fungal population in the WTBG remained the same from days 21 to 28, the reduction in C content can be attributed solely to rice straw decomposition. At day 42, the fungal population between NTBG and WTBG were not significantly different, while the C content in the WTBG was down to 28.63%, which is the lowest C content among the other samples (Table 3). This indicates that the difference in C content between the treatments was mainly due to the degradation of the rice straw. Differences in N content were also observed among the samples. A significant effect was observed on day 42, with the WTBG treatment having the highest N content at 1.1%.

**Table 3:** Decomposition parameters observed in litterbags.

Treatment	Carbon (%)	Nitrogen (%)	C/N Ratio	Mass loss (%)	Decomposition Rate (k)
Day 7					
NTSS	35.90^ns^	0.76^ns^	47.34^ns^	26.50^ns^	0.08^ns^
NTBG	35.40^ns^	0.64^ns^	55.17^ns^	32.36^ns^	0.11^ns^
WTSS	35.60^ns^	0.64^ns^	55.48^ns^	30.99^ns^	0.10^ns^
WTBG	34.50^ns^	0.81^ns^	42.86^ns^	28.34^ns^	0.09^ns^
Day 14					
NTSS	33.43^ns^	0.91^ns^	36.74^ns^	30.65^ns^	0.05^ns^
NTBG	32.43^ns^	0.65^ns^	49.64^ns^	32.99^ns^	0.06^ns^
WTSS	34.87^ns^	0.76^ns^	45.98^ns^	26.65^ns^	0.05^ns^
WTBG	33.63^ns^	0.64^ns^	52.42^ns^	25.71^ns^	0.05^ns^
Day 21					
NTSS	33.50^ns^	0.61^ns^	55.22^ns^	32.20^ab^	0.04^ab^
NTBG	33.23^ns^	0.69^ns^	48.28^ns^	37.67^a^	0.05^a^
WTSS	32.77^ns^	0.49^ns^	66.87^ns^	20.06^c^	0.02^c^
WTBG	34.70^ns^	0.56^ns^	61.96^ns^	28.29^bc^	0.03^b^
Day 28					
NTSS	34.30^a^	0.69^ns^	49.83^ns^	34.46^a^	0.03^a^
NTBG	31.10^b^	0.82^ns^	38.08^ns^	38.99^a^	0.04^a^
WTSS	33.83^a^	0.72^ns^	46.77^ns^	23.69^b^	0.02^b^
WTBG	29.77^c^	1.00^ns^	29.67^ns^	24.01^b^	0.02^b^
Day 42					
NTSS	34.40^a^	0.64^b^	53.60^a^	34.00^a^	0.020^ab^
NTBG	30.03^bc^	0.70^b^	43.43^a^	37.83^a^	0.024^a^
WTSS	32.30^ab^	0.69^b^	47.33^a^	20.77^b^	0.010^c^
WTBG	28.63^c^	1.10^a^	26.27^b^	31.23^a^	0.018^b^

*Means with the same letter are not significantly different (*p* < 0.05).

NTSS = no *Trichoderma* on soil surface; NTBG = no *Trichoderma* below ground; WTSS = with *Trichoderma* on soil surface; WTBG = with *Trichoderma* below ground.

Aside from determining weight loss, other researchers have used the C:N ratio as an indicator of decomposition rate^65, 66^. The C:N ratio of the plant litter is an indicator of the speed of decomposition and mineralization in soils. Crop residues with a narrow C:N ratio decompose faster than crop residues with a wide C:N ratio^67^. Crop residues with a high C:N ratio are also considered to be of low quality, whereas a low C:N ratio results in high-quality materials with a faster decomposition^68^. This is because residues with a low C:N ratio exhibit net N mineralization, while residues with a high C:N ratio exhibit immobilization^69–71^ In farming systems where the straw remains on the field after harvest, rapid decomposition is important to minimize negative effects caused by N immobilization on the following crops^72, 73^. The availability of other nutrients is also affected by the low quality of the rice straw, with a high C:N ratio, resulting in slow decomposition and mineralization of nutrients, particularly short-term availability of N and, to some extent, P^71^. The C:N ratio can be a reliable basis for measuring the decomposition of rice straw instead of only considering the results based on mass loss.

A computed C:N ratio of the plant litter is a better indicator of the decomposition rate since the values are expressed on a dry weight basis and the fluctuation in the values can be related to the *Trichoderma* population. In addition, it can indicate whether N will be mineralized or immobilized for the use by the succeeding crop. Rice straw is mostly made up of cellulose, resulting in its high C:N ratio and slow decomposition rate. A previous report mentioned that yield depression following straw incorporation can be mitigated by adding inorganic N^74^. In this study, the WTBG treatment had the lowest C/N Ratio at day 42, which was 26.27, compared with the NTBG, which had a high C/N ratio of 43.43. The C/N ratio of the WTBG was 39.53% lower than its uninoculated counterpart, even though no inorganic N had been added to the incorporated straw. If the fallow period of a farm is only 8 weeks, it is likely that the crops planted in areas with buried straw inoculated with *Trichoderma* will exhibit mineralization, while the uninoculated straws will show signs of immobilization.

## Conclusions

This study assessed the potential of a *Trichoderma*-based compost activator for the enhanced decomposition of incorporated rice straw by conducting experiments under both laboratory and field conditions. Results of the laboratory experiment indicated that the inoculant could increase decomposition rates under laboratory conditions, with an average of 16% higher amounts of CO_2_ released compared to uninoculated straw in sterile soil samples. In the field experiment, improved decomposition was observed in samples inoculated with *Trichoderma* and placed below ground (WTBG). From the initial value of around 35%, the C content in the WTBG was down to 28.63% after 42 days of incubation and was the lowest among treatments. The WTBG treatment also had the highest N content of 1.1%. The C:N ratio of the WTBG was only 26.27. This was 39.51% lower than the C:N ratio of the NTBG (no *Trichoderma* placed below ground), which was 43.43. At the end of the 6-week incubation period, the straw that was buried and inoculated with *Trichoderma* had the highest total fungal population, highest N content, lowest C content, and lowest C:N ratio, which are the conditions of an effective decomposition. These results confirmed that the *Trichoderma*-based compost activator can hasten the decomposition of incorporated rice straw. The increased fungal population in inoculated samples is an indication of enhanced biodiversity and the low C:N ratio promotes N mineralization.

While the improved chemical and biological properties in the incorporated straw indicated better decomposition, the changes in the physical conditions of the soil were not assessed in this study. Additional experiments may be needed to determine such properties since this information would be helpful in the field operations for the succeeding cropping period, which includes land and seedbed preparation. The use of additional amendments, such as manure and/or organic fertilizers or performing additional field operation activities, may also be tested to determine if the decomposition period can be shortened further.
