# Publisher Correction: Assessing the potential of a *Trichoderma*-based compost activator to hasten the decomposition of incorporated rice straw

**DOI:** 10.1038/s41598-022-05490-7

**Published:** 2022-01-25

**Authors:** Nolissa D. Organo, Shaira Mhel Joy M. Granada, Honey Grace S. Pineda, Joseph M. Sandro, Van Hung Nguyen, Martin Gummert

**Affiliations:** 1 Division of Soil Science, Agricultural Systems Institute, College of Agriculture and Food Science, University of the Philippines Los Baños, Laguna, Philippines; 2 Mechanization and Postharvest Cluster, Sustainable Impact Platform, International Rice Research Institute, 4030 Los Baños, Laguna Philippines

Correction to: *Scientific Reports* 10.1038/s41598-021-03828-1, published online 10 January 2022

The original version of this Article contained an error in Figure 1 where the x-axis was incorrectly numbered and the labels were illegible.

The original Figure 1 and accompanying legend appear below.

**Figure 1:**
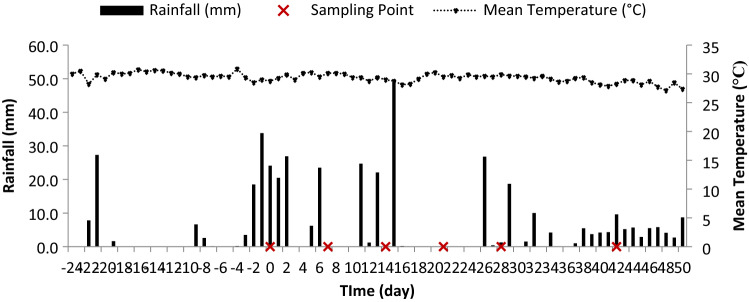
Average daily rainfall and temperature in the field during the experiment period.

The original Article has been corrected.

